# Preexisting statin therapy is not associated with reduced acute kidney injury following cardiac surgery: a retrospective analysis

**DOI:** 10.3389/fphar.2025.1613681

**Published:** 2025-05-30

**Authors:** Jia Wang, Chuzhu Huang, Yan Chen, Yilin Huang, Zhuomin Wu

**Affiliations:** The First Affiliated Hospital of Shantou University School of Medicine, Shantou, China

**Keywords:** CSA-AKI, statins, MIMIC-IV, cardiac surgery, AKI

## Abstract

**Background:**

Cardiac surgery-associated acute kidney injury (CSA-AKI) is one of the most prevalent forms of acute kidney injury (AKI) encountered in clinical practice, and its occurrence is significantly correlated with increased mortality and poor prognosis in patients. Although existing studies suggest that statins may influence the development of CSA-AKI through pleiotropic mechanisms, the findings from available studies and meta-analyses remain inconsistent. Therefore, the relationship between preexisting statin use and the risk of CSA-AKI development requires further investigation.

**Methods:**

This study employed a retrospective cohort analysis based on the MIMIC-IV database. Patients undergoing ascending aortic surgery, coronary artery bypass grafting (CABG), or heart valve surgery were included and categorized based on preexisting statin use. Multifactorial logistic regression models were utilized to assess the association between statin use and outcome metrics, adjusting for confounding variables. To further validate the results, propensity score matching (PSM), sensitivity analyses, and subgroup analyses were conducted.

**Results:**

A total of 4,783 patients were included, and the overall incidence of CSA-AKI was 30.02% (n = 1,436). Preliminary analysis showed that the incidence of AKI was significantly higher in the statin use group than in the non-use group (34.06% vs. 29.23%, P = 0.007). In the uncorrected model, statin use was associated with an elevated risk of AKI (OR = 1.25, 95% CI: 1.06–1.47); however, after multifactorial correction, the association was not statistically significant (OR = 1.00, 95% CI: 0.00-Inf, P = 1.000). Similarly, in the uncorrected model, statin use was associated with increased in-hospital mortality (OR = 1.28, 95% CI: 1.01–1.62) and ICU mortality (OR = 1.36, 95% CI: 1.07–1.72); however, after multifactorial correction, statin use was not significantly associated with in-hospital mortality (HR, 1.19; 95% CI, 0.92–1.53; P = 0.184) and ICU mortality (HR, 1.21; 95% CI, 0.94–1.55; P = 0.147) in the corrected model. PSM analysis (1:1 matching) further confirmed these findings (AKI: OR = 1.05, P = 0.621; in-hospital mortality: HR = 1.13, P = 0.438; ICU mortality: HR = 1.18, P = 0.299). None of the subgroup analyses (stratified by statin dose, AKI severity, and type of surgery) revealed significant interactions. Before PSM, no statistically significant differences were observed in 30-day (p = 0.126), 60-day (p = 0.372), or 90-day mortality (p = 0.652). After PSM, the mortality rates remained comparable between groups at all time points (30-day p = 0.297; 60-day p = 0.837; 90-day p = 0.966).

**Conclusion:**

Preexisting statin use was not significantly associated with the risk of developing CSA-AKI, in-hospital mortality, or ICU mortality after appropriate correction for confounding variables. Similarly, no significant associations were observed for 30-day, 60-day, or 90-day mortality outcomes. Sensitivity analyses and subgroup analyses consistently supported this conclusion, suggesting that statin use may not significantly impact clinical outcomes in patients undergoing cardiac surgery.

## 1 Introduction

Acute kidney injury (AKI) represents a significant global public health challenge, particularly among patients in intensive care units (ICU) (Bao et al., 2018; Zou et al., 2022). As an independent risk factor for various adverse outcomes, AKI is not only strongly associated with the development of end-stage renal disease (ESRD), but it also significantly increases short-term morbidity and mortality, as well as the need for dialysis (Teo and Endre, 2017). In the context of cardiac surgery, the incidence of cardiac surgery-associated AKI (CSA-AKI) ranges from 5% to 81% (Wang and Bellomo, 2017; Priyanka et al., 2021; Wang et al., 2021), which independently prolongs hospitalization (Chertow et al., 2005), escalates healthcare costs, and is closely linked to increased in-hospital mortality and decreased long-term survival (Li et al., 2011; Bihorac et al., 2009). Notably, severe AKI is associated with an eightfold increase in 30-day mortality (Chertow et al., 1998), underscoring its critical impact on patient prognosis. Given this evidence, international guidelines emphasize the prevention of CSA-AKI as a core component of perioperative management in cardiac surgery (Macedo and Mehta, 2015). Consequently, the exploration of effective prevention and treatment strategies remains a key focus of current research in nephrology and critical care medicine (Wang and Bellomo, 2017).

Statins, known as 3-Hydroxy-3-methylglutaryl coenzyme A (HMG-CoA) reductase inhibitors are among the most commonly prescribed medications worldwide (Santodomingo-Garzón et al., 2006). In addition to reducing the risk of cardiovascular mortality by lowering serum cholesterol levels (Rosenson, 2006), statins exhibit significant pleiotropic effects, including anti-inflammatory, anti-thrombotic, and immunomodulatory properties (Novack et al., 2006; Terblanche et al., 2007; Wang et al., 2008). Statins remain the foundational agents in cardiovascular prevention (López-Miranda and Pedro-Botet, 2021), effectively reducing atherosclerotic cardiovascular disease (ASCVD) risk—including coronary heart disease and stroke—in both primary and secondary prevention contexts (Ray, 2024). Their proven benefits extend to diverse and complex populations, such as individuals with chronic kidney disease (CKD), HIV infection, and metabolic-associated steatotic liver disease (MASLD) (Safarova et al., 2025). Animal studies have confirmed that statins may exert nephroprotective effects by attenuating oxidative stress and improving endothelial function (Sabbatini et al., 2004; Gueler et al., 2002). However, the conclusions of clinical studies regarding the relationship between statins and AKI remain controversial. The protective effect of statins may vary depending on the type of AKI. Some studies suggest that their neuroprotective (Kivipelto et al., 2005), immunomodulatory (Greenwood et al., 2006), and cellular senescence-delaying (Brouilette et al., 2007) effects may reduce the incidence of CSA-AKI (Tu et al., 2021), particularly in preventing CSA-AKI by decreasing the damage marker (Molnar et al., 2014). Conversely, other studies have failed to confirm such protective effects (Park et al., 2016; Zheng et al., 2016). Therefore, elucidating the potential inhibitory effect of early statin application on CSA-AKI necessitates further high-quality clinical evidence.

The aim of this study was to systematically assess the effect of preexisting statin use on the risk of postoperative AKI in adult ICU patients following cardiac surgery through a retrospective cohort analysis. Additionally, we sought to analyze the correlation between statin use and clinical outcomes, such as in-hospital mortality and ICU mortality. We also explored the quantitative and qualitative relationship between statin dosage and AKI occurrence, the association characteristics of different severities of AKI (based on KDIGO staging criteria), and the differential performance across various types of cardiac surgery, including coronary artery bypass grafting, valve surgery, and aortic surgery.

## 2 Materials and methods

### 2.1 Sources of data

This retrospective study utilized health-related data obtained from the MIMIC-IV (version 3.1) database, a comprehensive and extensive resource developed and managed by the MIT Computational Physiology Laboratory. This database comprises high-quality medical records of patients admitted to the intensive care units of the Beth Israel Deaconess Medical Center (Johnson et al., 2023). Jia Wang, one of the authors, collected clinical data from the MIMIC database (certification number: 42,257,067), including patient demographic information, laboratory findings, and medication usage. This project adhered to the principles of the Helsinki Declaration, and approval from the ethics committee was not required due to participant anonymity and data standardization within the database.

### 2.2 Study population

**FIGURE 1:**
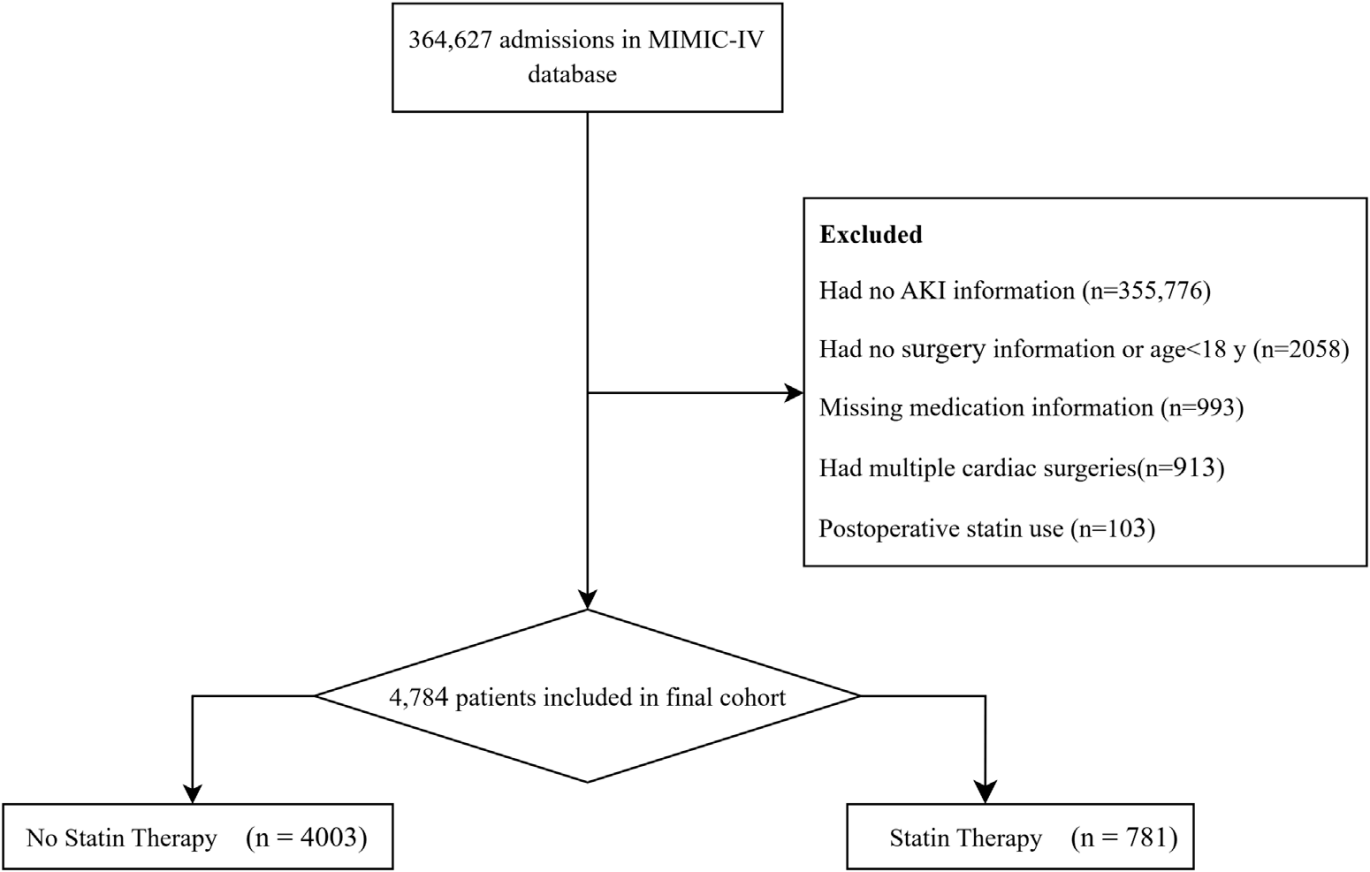
Flowchart of the patient enrollment process.

This study screened all adult patients (≥18 years) admitted to the ICU following cardiac surgery. Inclusion criteria encompassed patients undergoing coronary artery bypass grafting (CABG), valve surgery, or aortic surgery (including combined procedures: e.g., CABG + valve surgery). According to predetermined exclusion criteria, patients were excluded for the following reasons: (1) lack of data on AKI assessment within 48 h of ICU admission, (2) missing information on medication use, and (3) use of statins after surgery (regardless of preexisting use). For patients who underwent multiple cardiac surgeries, only data from their first surgery were included. After rigorous screening, a total of 4,784 patients were enrolled in the study cohort, comprising 781 in the statin therapy group and 4,003 in the no statin therapy group (Figure 1).

### 2.3 Data collection and definitions

Structured Query Language (SQL) was employed to extract data using Navicat Premium software (version 15) based on unique patient identifiers (e.g., stay_id). We collected demographic information, including age, gender, body mass index (BMI), and race. Additionally, laboratory indices such as the SOFA score, APS III, and APACHE II were extracted within the first 24 h of ICU admission. Comorbidities, including hypertension, diabetes, chronic kidney disease, heart failure, myocardial infarction, and chronic obstructive pulmonary disease (COPD), were identified using ICD-9/10 codes. Medication use, including statins, neuromuscular blockers, glucocorticoids, aspirin, and antibiotics, was documented. Laboratory variables, including hematocrit, hemoglobin, platelet count, red cell distribution width (RDW), electrolytes (sodium, potassium, calcium, chloride), glucose, lactate, pH, PCO_2_, and PO_2_, were collected.

Patients diagnosed with AKI were staged according to the KDIGO criteria (Wang and Bellomo, 2017). Stage 1 was classified as Mild AKI, defined by a serum creatinine (SCr) increase to 1.5–1.9 times baseline or a rise of ≥0.3 mg/dL (≥26.5 μmol/L), or urine output <0.5 mL/(kg·h) for 6–12 h. Stages 2 and 3 were classified as Severe AKI, with Stage 2 defined by an SCr increase to 2.0–2.9 times baseline or urine output <0.5 mL/(kg·h) for ≥12 h, and Stage 3 defined by an SCr increase to 3 times baseline, SCr ≥4.0 mg/dL (≥353.6 μmol/L), initiation of renal replacement therapy, or urine output <0.3 mL/(kg·h) for ≥24 h (or anuria for ≥12 h).

All statins were converted to equivalent doses of simvastatin (Chou et al., 2022) and categorized at the median to construct high-dose (≥40 mg) and low-dose (<40 mg) statin groups.

### 2.4 Outcomes

In this study, the primary outcome measured was the incidence of AKI following cardiac surgery, based on KDIGO criteria. The secondary outcomes investigated included the association between in-hospital mortality and ICU mortality, the impact of different doses of statins on AKI, variations in the effect of statins across different stages of AKI, and differences in statin effects among various surgical procedures regarding the incidence of AKI.

### 2.5 Statistical analysis

In this study, continuous variables did not follow a normal distribution and were therefore presented as medians with interquartile ranges (IQR). Categorical variables were expressed as frequencies and percentages. Baseline characteristics of statin-treated and untreated patients were compared using the Mann-Whitney test for continuous variables and the Chi-square test for categorical variables. Variables with less than 20% missing data were retained. All missing values were addressed using multiple imputation methods in SPSS version 27.

Multivariable logistic regression models were constructed to adjust for potential confounders. Cox proportional hazards models were employed to estimate the associations of statin use with survival rates in both hospital and ICU settings. Odds ratios (ORs) and 95% confidence intervals (CIs) were calculated. Model 1 was unadjusted, while Model 2 adjusted for age, gender, race, SOFA score, APS III score, and APACHE II score. Model 3 was further adjusted for comorbidities, including hepatitis, cerebrovascular accident, chronic kidney disease, cancer, T2DM, T1DM, hyperlipidemia, chronic bronchitis, heart failure, ischemic heart disease, and COPD. Model 4, the fully adjusted model, included all variables from Model 3, as well as laboratory variables (hematocrit, hemoglobin, red cell distribution width (RDW), red blood cell count, white blood cell count, anion gap, and chloride).

To further control for confounding, propensity score matching (PSM) was performed using a 1:1 nearest neighbor matching approach with a caliper width of 0.25. The variables selected for PSM were those with a P-value of less than 0.05 in the baseline table. Standardized Mean Difference (SMD) was used to evaluate the similarity of the distribution of covariates in the matched samples. Logistic regression was then performed on the matched cohort to assess the association between statin use and AKI incidence. Cox proportional hazards models were again utilized to estimate the associations of statin use with survival rates in both hospital and ICU settings.

Subgroup analyses were conducted to explore potential effect modification by statin dosage (low vs. high), AKI severity (mild vs. severe), and surgery type (aortic surgery, coronary bypass, valve surgery, combined). The subgroup analysis prior to PSM included all variables with a P-value of less than 0.05 from the baseline analysis. The subgroup analysis following PSM served as a sensitivity analysis to further evaluate the association between statin use and the incidence of AKI in matched subgroups, defined by statin dosage, AKI severity, and type of cardiac surgery. The results of the subgroup analyses before and after PSM were presented together in the same table. For the subgroup analysis after PSM, covariates with a P-value of less than 0.05, including white blood cell count and calcium, were incorporated.

All statistical analyses were performed using R version 4.4.1 and SPSS version 27. A two-sided P-value of less than 0.05 was considered statistically significant.

## 3 Results

### 3.1 Patient characteristics

**FIGURE 2:**
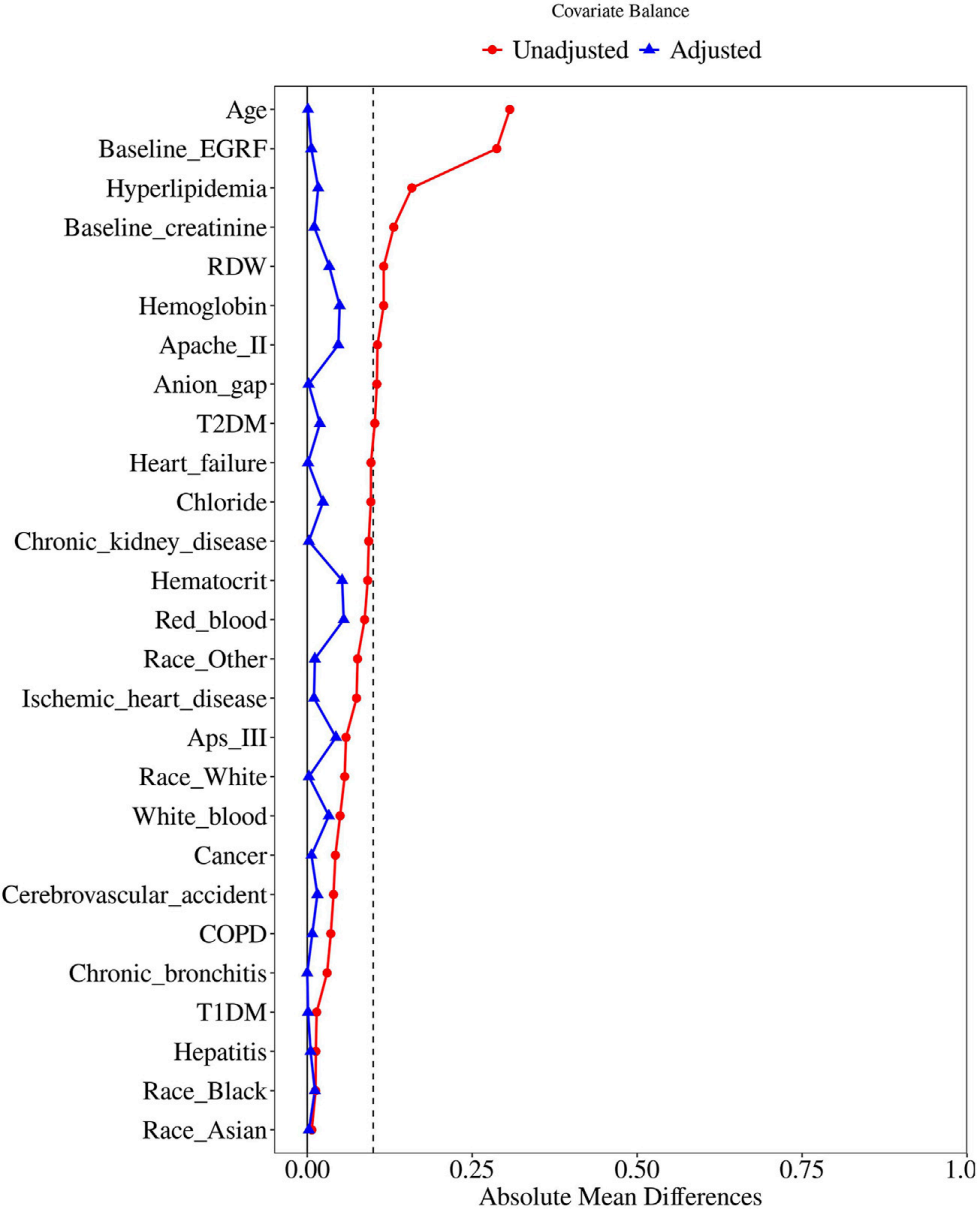
Love plot of standardized mean differences for covariate balance.

In this study, 4,784 patients were divided into two groups: the statin therapy group and the no statin therapy group (Table 1). Significant differences between the two groups were observed prior to PSM analysis, including patient characteristics (age, race: Asian, Black, Other, White), laboratory indices (APS III, APACHE II), comorbidities (hepatitis, cerebrovascular accident, chronic kidney disease, cancer, T2DM, T1DM, hyperlipidemia, chronic bronchitis, heart failure, ischemic heart disease, and COPD), baseline measurements (baseline creatinine, baseline eGFR), and laboratory variables (hematocrit, hemoglobin, RDW, red blood cell count, white blood cell count, anion gap, and chloride). Subsequently, 781 patients in the statin therapy group were matched with 781 patients in the no statin therapy group through 1:1 matching (Figure 2). After matching, among the baseline characteristics of the two groups, only the p-values for white blood and calcium were less than 0.05.

**TABLE 1:** Baseline characteristics of patients before and after propensity score matching.

Variables	Before PSM				After PSM			
	Total (n = 4,784)	No statin therapy (n = 4,003)	Statin therapy (n = 781)	P-value	Total (n = 1,562)	No statin therapy (n = 781)	Statin therapy (n = 781)	P-value
Patient characteristics								
Age, M (Q_1_, Q_3_)	70.00 (61.00, 77.00)	69.00 (61.00, 77.00)	72.00 (64.00, 79.00)	<0.001	72.00 (65.00, 79.00)	72.00 (65.00, 79.00)	72.00 (64.00, 79.00)	0.951
Bmi, M (Q_1_, Q_3_)	29.31 (25.87, 33.34)	29.27 (25.81, 33.35)	29.40 (26.27, 33.21)	0.788	29.49 (26.20, 33.35)	29.53 (26.01, 33.39)	29.40 (26.27, 33.21)	0.838
Gender, n (%)				0.225				0.869
F	1,396 (29.18)	1,154 (28.83)	242 (30.99)		481 (30.79)	239 (30.60)	242 (30.99)	
M	3,388 (70.82)	2,849 (71.17)	539 (69.01)		1,081 (69.21)	542 (69.40)	539 (69.01)	
Race, n (%)				<0.001				0.723
Asian	95 (1.99)	75 (1.87)	20 (2.56)		38 (2.43)	18 (2.30)	20 (2.56)	
Black	274 (5.73)	221 (5.52)	53 (6.79)		97 (6.21)	44 (5.63)	53 (6.79)	
Other	924 (19.31)	823 (20.56)	101 (12.93)		211 (13.51)	110 (14.08)	101 (12.93)	
White	3,491 (72.97)	2,884 (72.05)	607 (77.72)		1,216 (77.85)	609 (77.98)	607 (77.72)	
Surgery type				0.860				0.397
Aortic surgery	1,154 (24.12)	967 (24.16)	187 (23.94)		352 (22.54)	165 (21.13)	187 (23.94)	
Coronary bypass	2,132 (44.57)	1,774 (44.32)	358 (45.84)		746 (47.76)	388 (49.68)	358 (45.84)	
Valve surgery	1,256 (26.25)	1,057 (26.41)	199 (25.48)		395 (25.29)	196 (25.10)	199 (25.48)	
Combined	242 (5.06)	205 (5.12)	37 (4.74)		69 (4.42)	32 (4.10)	37 (4.74)	
Laboratory Index								
Sofa, M (Q_1_, Q_3_)	5.00 (3.00, 7.00)	5.00 (3.00, 7.00)	5.00 (4.00, 7.00)	0.311	5.00 (4.00, 7.00)	5.00 (3.71, 7.00)	5.00 (4.00, 7.00)	0.707
Aps III, M (Q_1_, Q_3_)	37.00 (29.00, 49.00)	37.00 (29.00, 49.00)	38.00 (30.00, 53.00)	0.028	38.00 (30.00, 50.00)	39.00 (30.00, 49.00)	38.00 (30.00, 53.00)	0.660
Apache II, M (Q_1_, Q_3_)	18.00 (15.00, 23.00)	18.00 (15.00, 23.00)	19.00 (15.00, 24.00)	0.006	19.00 (15.00, 23.00)	19.00 (15.00, 23.00)	19.00 (15.00, 24.00)	0.607
Comorbidities								
Hypertension, n (%)	2,285 (47.76)	1924 (48.06)	361 (46.22)	0.346	695 (44.49)	334 (42.77)	361 (46.22)	0.169
Liver cirrhosis, n (%)	118 (2.47)	100 (2.50)	18 (2.30)	0.750	34 (2.18)	16 (2.05)	18 (2.30)	0.729
Hepatitis, n (%)	132 (2.76)	119 (2.97)	13 (1.66)	0.041	30 (1.92)	17 (2.18)	13 (1.66)	0.461
Pulmonary tuberculosis, n (%)	10 (0.21)	9 (0.22)	1 (0.13)	0.910	2 (0.13)	1 (0.13)	1 (0.13)	1.000
Pneumonia, n (%)	504 (10.54)	426 (10.64)	78 (9.99)	0.586	165 (10.56)	87 (11.14)	78 (9.99)	0.459
Cerebrovascular accident, n (%)	398 (8.32)	307 (7.67)	91 (11.65)	<0.001	170 (10.88)	79 (10.12)	91 (11.65)	0.330
Chronic kidney disease, n (%)	1,140 (23.83)	893 (22.31)	247 (31.63)	<0.001	496 (31.75)	249 (31.88)	247 (31.63)	0.913
Cancer, n (%)	699 (14.61)	557 (13.91)	142 (18.18)	0.002	279 (17.86)	137 (17.54)	142 (18.18)	0.741
T2DM, n (%)	1,722 (35.99)	1,374 (34.32)	348 (44.56)	<0.001	711 (45.52)	363 (46.48)	348 (44.56)	0.446
T1DM, n (%)	90 (1.88)	66 (1.65)	24 (3.07)	0.007	47 (3.01)	23 (2.94)	24 (3.07)	0.882
Hyperlipidemia, n (%)	3,310 (69.19)	2,666 (66.60)	644 (82.46)	<0.001	1,301 (83.29)	657 (84.12)	644 (82.46)	0.378
Chronic bronchitis, n (%)	492 (10.28)	392 (9.79)	100 (12.80)	0.011	200 (12.80)	100 (12.80)	100 (12.80)	1.000
Heart failure, n (%)	1,659 (34.68)	1,325 (33.10)	334 (42.77)	<0.001	669 (42.83)	335 (42.89)	334 (42.77)	0.959
Myocardial infarction, n (%)	1,055 (22.05)	879 (21.96)	176 (22.54)	0.722	371 (23.75)	195 (24.97)	176 (22.54)	0.259
Ischemic heart disease, n (%)	3,474 (72.62)	2,858 (71.40)	616 (78.87)	<0.001	1,240 (79.39)	624 (79.90)	616 (78.87)	0.617
COPD, n (%)	592 (12.37)	472 (11.79)	120 (15.36)	0.006	234 (14.98)	114 (14.60)	120 (15.36)	0.671
Lab variables								
Serum creatinine, M (Q_1_, Q_3_)	1.01 (0.82, 1.39)	1.00 (0.81, 1.36)	1.08 (0.85, 1.55)	<0.001	1.09 (0.85, 1.56)	1.09 (0.84, 1.57)	1.08 (0.85, 1.55)	0.935
Hemoglobin, M (Q_1_, Q_3_)	9.69 (8.82, 10.78)	9.70 (8.83, 10.81)	9.60 (8.79, 10.63)	0.064	9.55 (8.78, 10.62)	9.53 (8.74, 10.60)	9.60 (8.79, 10.63)	0.608
EGRF, M (Q_1_, Q_3_)	81.99 (70.69, 91.93)	82.83 (71.62, 92.50)	78.00 (67.01, 88.20)	<0.001	78.12 (66.52, 88.16)	78.26 (66.03, 87.61)	78.00 (67.01, 88.20)	0.890
Medications								
Neuromuscular blocker, n (%)	162 (3.39)	141 (3.52)	21 (2.69)	0.239	47 (3.01)	26 (3.33)	21 (2.69)	0.459
Glucocorticoid, n (%)	550 (11.50)	450 (11.24)	100 (12.80)	0.210	192 (12.29)	92 (11.78)	100 (12.80)	0.538
Aspirin, n (%)	4,065 (84.97)	3,395 (84.81)	670 (85.79)	0.485	1,342 (85.92)	672 (86.04)	670 (85.79)	0.884
Antibiotics, n (%)	4,361 (91.16)	3,641 (90.96)	720 (92.19)	0.267	1,431 (91.61)	711 (91.04)	720 (92.19)	0.411
Lab variables								
Hematocrit, M (Q_1_, Q_3_)	28.70 (25.20, 32.31)	28.80 (25.30, 32.40)	28.20 (24.90, 31.90)	0.042	28.40 (24.90, 32.10)	28.40 (24.80, 32.30)	28.20 (24.90, 31.90)	0.603
Hemoglobin, M (Q_1_, Q_3_)	9.40 (8.20, 10.70)	9.40 (8.20, 10.70)	9.20 (8.10, 10.50)	0.008	9.30 (8.01, 10.51)	9.30 (8.00, 10.60)	9.20 (8.10, 10.50)	0.583
Platelet, M (Q_1_, Q_3_)	143.00 (111.00, 184.82)	143.00 (111.00, 185.00)	141.00 (111.00, 184.00)	0.437	143.50 (111.00, 185.00)	146.00 (113.00, 187.00)	141.00 (111.00, 184.00)	0.272
RDW, M (Q_1_, Q_3_)	13.60 (12.90, 14.90)	13.60 (12.90, 14.89)	13.80 (13.00, 15.30)	<0.001	13.80 (13.00, 15.10)	13.70 (13.00, 15.00)	13.80 (13.00, 15.30)	0.366
Red blood, M (Q_1_, Q_3_)	3.13 (2.72, 3.57)	3.14 (2.73, 3.58)	3.08 (2.70, 3.48)	0.040	3.10 (2.68, 3.53)	3.11 (2.67, 3.57)	3.08 (2.70, 3.48)	0.462
White blood, M (Q_1_, Q_3_)	12.20 (8.80, 15.90)	12.30 (8.90, 16.00)	11.60 (8.30, 15.40)	0.001	11.90 (8.70, 15.60)	12.20 (9.00, 15.80)	11.60 (8.30, 15.40)	0.025
Anion gap, M (Q_1_, Q_3_)	12.00 (9.00, 14.00)	12.00 (9.00, 14.00)	12.00 (10.00, 15.00)	0.009	12.00 (10.00, 15.00)	12.00 (10.00, 15.00)	12.00 (10.00, 15.00)	0.774
Calcium, M (Q_1_, Q_3_)	8.30 (7.98, 8.70)	8.30 (8.00, 8.66)	8.30 (7.90, 8.70)	0.547	8.30 (8.00, 8.70)	8.34 (8.00, 8.70)	8.30 (7.90, 8.70)	0.013
Chloride, M (Q_1_, Q_3_)	107.00 (104.00, 109.00)	107.00 (104.00, 109.00)	107.00 (103.00, 109.00)	0.041	106.00 (103.00, 109.00)	106.00 (103.00, 109.00)	107.00 (103.00, 109.00)	0.466
Glucose, M (Q_1_, Q_3_)	123.00 (107.00, 144.00)	123.00 (107.00, 144.00)	123.00 (107.00, 147.00)	0.189	124.00 (107.00, 148.00)	124.00 (107.00, 149.00)	123.00 (107.00, 147.00)	0.908
Potassium, M (Q_1_, Q_3_)	4.40 (4.00, 4.70)	4.40 (4.00, 4.70)	4.40 (4.00, 4.70)	0.595	4.40 (4.00, 4.70)	4.40 (4.00, 4.73)	4.40 (4.00, 4.70)	0.308
Sodium, M (Q_1_, Q_3_)	138.00 (136.00, 140.00)	138.00 (136.00, 140.00)	138.00 (136.00, 140.00)	0.223	138.00 (136.00, 140.00)	138.00 (136.00, 140.00)	138.00 (136.00, 140.00)	0.636
CO2, M (Q_1_, Q_3_)	25.00 (23.49, 27.00)	25.00 (23.35, 27.00)	25.00 (24.00, 27.00)	0.283	25.00 (23.65, 27.00)	25.00 (23.00, 27.00)	25.00 (24.00, 27.00)	0.416
Free calcium, M (Q_1_, Q_3_)	1.13 (1.08, 1.20)	1.13 (1.08, 1.20)	1.13 (1.07, 1.20)	0.168	1.13 (1.07, 1.19)	1.13 (1.07, 1.19)	1.13 (1.07, 1.20)	0.830
Lactate, M (Q_1_, Q_3_)	1.90 (1.40, 2.60)	1.90 (1.40, 2.60)	1.90 (1.30, 2.50)	0.080	1.90 (1.40, 2.60)	1.90 (1.40, 2.60)	1.90 (1.30, 2.50)	0.290
PCO2, M (Q_1_, Q_3_)	41.00 (37.00, 45.00)	41.00 (37.00, 45.00)	41.00 (37.00, 45.00)	0.559	41.00 (37.00, 45.00)	41.00 (37.00, 45.00)	41.00 (37.00, 45.00)	0.311
PH, M (Q_1_, Q_3_)	7.39 (7.35, 7.43)	7.39 (7.35, 7.43)	7.39 (7.34, 7.43)	0.505	7.39 (7.35, 7.43)	7.39 (7.35, 7.43)	7.39 (7.34, 7.43)	0.439
PO2, M (Q_1_, Q_3_)	287.00 (183.00, 352.00)	286.00 (182.00, 351.00)	290.00 (186.52, 353.00)	0.484	289.05 (184.00, 353.00)	289.00 (180.23, 352.00)	290.00 (186.52, 353.00)	0.645
Dosage Effects								
Low-dose, n (%)	314 (6.56)	0 (0.00)	314 (40.20)	<0.001	314 (20.10)	0 (0.00)	314 (40.20)	<0.001
High-dose, n (%)	467 (9.76)	0 (0.00)	467 (59.80)	<0.001	467 (29.90)	0 (0.00)	467 (59.80)	<0.001
Duration of statin use, (hours)	0.00 (0.00, 0.00)	0.00 (0.00, 0.00)	97.00 (17.00, 166.00)	<0.001	0.00 (0.00, 119.75)	0.00 (0.00, 0.00)	120.00 (52.00, 190.00)	<0.001
Primary Outcomes								
AKI, n (%)				0.007				0.708
No	3,348 (69.98)	2,833 (70.77)	515 (65.94)		1,037 (66.39)	522 (66.84)	515 (65.94)	
Yes	1,436 (30.02)	1,170 (29.23)	266 (34.06)		525 (33.61)	259 (33.16)	266 (34.06)	
Mild AKI, n (%)	897 (18.75)	743 (18.56)	154 (19.72)	0.448	314 (20.10)	160 (20.49)	154 (19.72)	0.705
Severe AKI, n (%)	539 (11.27)	427 (10.67)	112 (14.34)	0.003	211 (13.51)	99 (12.68)	112 (14.34)	0.336
RRT, n (%)	207 (4.33)	161 (4.02)	46 (5.89)	0.019	79 (5.06)	33 (4.23)	46 (5.89)	0.133
Duration of RRT, M (Q_1_, Q_3_)	5.00 (2.50, 9.00)	5.00 (3.00, 9.00)	4.50 (2.00, 8.75)	0.577	5.00 (2.50, 8.00)	6.00 (3.00, 8.00)	4.50 (2.00, 8.75)	0.730
Secondary outcomes								
Dead, n (%)	778 (16.26)	607 (15.16)	171 (21.90)	<0.001	310 (19.85)	139 (17.80)	171 (21.90)	0.042
Hosp day, M (Q_1_, Q_3_)	12.83 (6.84, 23.06)	13.52 (7.11, 23.87)	11.00 (5.83, 19.75)	0.013	11.87 (6.11, 20.59)	13.15 (7.07, 22.20)	11.00 (5.83, 19.75)	0.178
ICU day, M (Q_1_, Q_3_)	3.37 (1.60, 7.87)	3.74 (1.69, 9.33)	2.84 (1.36, 5.70)	0.004	3.01 (1.38, 6.15)	3.44 (1.39, 7.46)	2.84 (1.36, 5.70)	0.320
30-day mortality, n (%)	169 (3.53)	132 (3.30)	37 (4.74)	0.126	62 (3.97)	25 (3.20)	37 (4.74)	0.297
60-day mortality, n (%)	208 (4.35)	167 (4.17)	41 (5.25)	0.372	77 (4.93)	36 (4.61)	41 (5.25)	0.837
90-day mortality, n (%)	229 (4.79)	187 (4.67)	42 (5.38)	0.652	82 (5.25)	40 (5.12)	42 (5.38)	0.966

BMI, body mass index; SOFA, sequential organ failure assessment; APS III, Acute Physiology Score III; APACHE II, Acute Physiology and Chronic Health Evaluation II; T2DM, Type 2 diabetes mellitus; T1DM, Type 1 diabetes mellitus; COPD, chronic obstructive pulmonary disease; EGRF, epidermal growth factor receptor; RDW, red cell distribution width; PCO_2_, partial pressure of carbon dioxide; PO_2_, partial pressure of oxygen; RRT, renal replacement therapy; Z, Mann-Whitney test; χ^2^, Chi-square test; M, Median, Q_1_, 1st Quartile; Q_3_, 3st Quartile.

### 3.2 Multivariable logistic and cox regression analysis

#### 3.2.1 Primary outcome: AKI

In this study, statins were administered for a median duration of approximately 7 days (167 h) prior to surgery in the pre-PSM cohort, and 7.3 days (176 h) in the post-PSM cohort (Table 1). Among patients receiving preexisting statin therapy, the AKI incidence was significantly higher compared to non-statin users (34.06% vs. 29.23%) (Table 2). In the unadjusted logistic regression model (Model 1), statin therapy was significantly associated with an increased risk of AKI (OR, 1.25; 95% CI, 1.06–1.47; P = 0.007). However, after adjusting for demographic parameters (Model 2), comorbidities (Model 3), and laboratory variables (Model 4), this association was no longer significant (OR, 1.00; 95% CI, 0.00–Inf; P = 1.000 for all adjusted models).

**TABLE 2:** Multivariable-adjusted associations between preexisting statin use and primary/secondary outcomes across different models.

Variables	No. of patients with event (%)		Model 1		Model 2		Model 3		Model 4	
	Statin users	Statin nonusers	OR (95%CI)	P-value	OR (95%CI)	P-value	OR (95%CI)	P-value	OR (95%CI)	P-value
Primary outcome										
AKI	1,170 (29.23)	266 (34.06)	1.25 (1.06 ∼ 1.47)	0.007	1.00 (0.00 ∼ Inf)	1.000	1.00 (0.00 ∼ Inf)	1.000	1.00 (0.00 ∼ Inf)	1.000
Secondary outcomes										
In-Hospital Mortality	607 (15.16)	171 (21.90)	1.28 (1.01 ∼ 1.62)	0.042	1.28 (1.01 ∼ 1.63)	0.040	1.18 (0.92 ∼ 1.51)	0.190	1.19 (0.92 ∼ 1.53)	0.184
In-ICU Mortality			1.36 (1.07 ∼ 1.72)	0.011	1.35 (1.06 ∼ 1.71)	0.013	1.24 (0.97 ∼ 1.59)	0.091	1.21 (0.94 ∼ 1.55)	0.147

OR: Odds Ratio, CI: Confidence IntervalModel1: Unadjusted model. Model 2: Adjusted for Age, Gender, Race, Sofa, Aps III, Apache II., Model 3: Futher adjusted for Comorbidities (Hepatitis; Cerebrovascular accident; Chronic kidney disease; Cancer; T2DM; T1DM; hyperlipidemia; Chronic bronchitis; Heart failure; Ischemic heart disease; COPD). Model 4: Futher adjusted for Lab variables (Hematocrit; Hemoglobin; RDW; red blood; White blood; Anion gap; Chloride).

#### 3.2.2 Secondary outcomes: in-hospital mortality and In-ICU mortality

For in-hospital mortality, Cox regression analysis revealed that statin therapy was associated with a significantly higher risk in the unadjusted model (HR, 1.28; 95% CI, 1.01–1.62; P = 0.042) and after adjusting for demographic parameters (Model 2: HR, 1.28; 95% CI, 1.01–1.63; P = 0.040). However, further adjustment for comorbidities and laboratory variables attenuated this association, rendering it non-significant (Model 3: HR, 1.18; 95% CI, 0.92–1.51; P = 0.190; Model 4: HR, 1.19; 95% CI, 0.92–1.53; P = 0.184).

Similarly, for in-ICU mortality, statin therapy showed a significant association in the unadjusted Cox model (HR, 1.36; 95% CI, 1.07–1.72; P = 0.011) and after adjusting for demographic parameters (Model 2: HR, 1.35; 95% CI, 1.06–1.71; P = 0.013). However, this association was no longer significant after further adjustment for comorbidities and laboratory variables (Model 3: HR, 1.24; 95% CI, 0.97–1.59; P = 0.091; Model 4: HR, 1.21; 95% CI, 0.94–1.55; P = 0.147).

### 3.3 Sensitivity analysis

**FIGURE 3:**
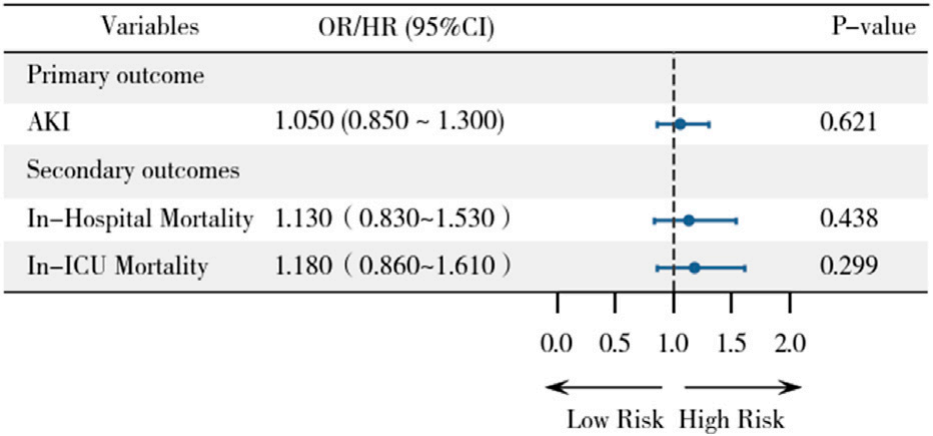
Forest plot demonstrating associations between statin use and primary/secondary outcomes after PSM.

Variables that remained statistically significant (p < 0.05) after propensity score matching were included as covariates in the final analysis (white blood and calcium). Logistic regression analysis after PSM showed no significant association between statin therapy and AKI incidence (OR, 1.05; 95% CI, 0.85–1.30; P = 0.621) (Figure 3). This result is consistent with the findings from the multivariable logistic regression before PSM, suggesting that statin use is not significantly associated with an increased or decreased risk of AKI.

Similarly, no significant associations were observed between statin therapy and in-hospital mortality (HR, 1.13; 95% CI, 0.83–1.53; P = 0.438) or ICU mortality (HR, 1.18; 95% CI, 0.86–1.61; P = 0.299) after PSM. These findings further support the robustness of our primary results, indicating that statin therapy does not significantly impact mortality outcomes in this population.

### 3.4 Subgroup analysis

**FIGURE 4:**
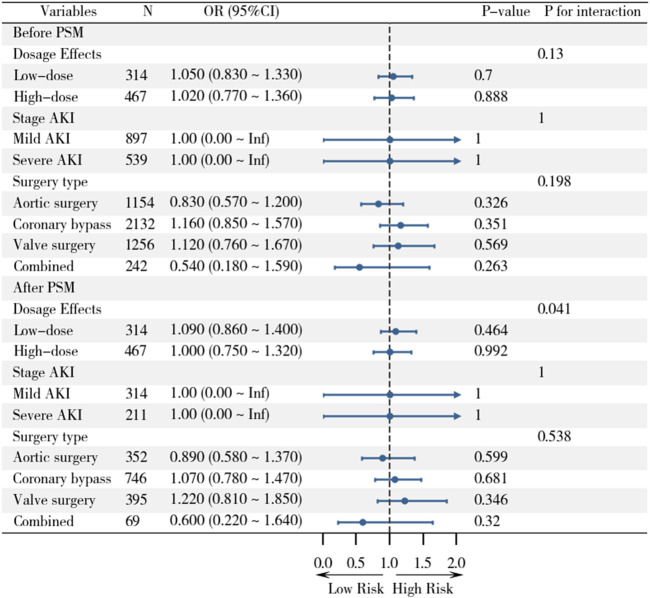
Subgroup analyses of statin effects on clinical outcomes across different populations before and after PSM.

Subgroup analyses were performed to explore potential effect modifications by statin dose, AKI severity, and surgery type (Figure 4). In the before PSM analysis, no significant interactions were observed between statin therapy and AKI incidence across subgroups. For statin dose, low-dose statin use (OR, 1.05; 95% CI, 0.83–1.33; P = 0.700) and high-dose statin use (OR, 1.02; 95% CI, 0.77–1.36; P = 0.888) showed no significant association with AKI. Similarly, no significant differences were found between mild AKI (OR, 1.00; 95% CI, 0.00–Inf; P = 1.000) and severe AKI (OR, 1.00; 95% CI, 0.00–Inf; P = 1.000). When stratified by surgery type, the association between statin therapy and AKI remained non-significant for aortic surgery (OR, 0.83; 95% CI, 0.57–1.20; P = 0.326), coronary bypass (OR, 1.16; 95% CI, 0.85–1.57; P = 0.351), valve surgery (OR, 1.12; 95% CI, 0.76–1.67; P = 0.569), and combined surgery (OR, 0.54; 95% CI, 0.18–1.59; P = 0.263).

After PSM, the results remained consistent. Low-dose statin use (OR, 1.09; 95% CI, 0.86–1.40; P = 0.464) and high-dose statin use (OR, 1.00; 95% CI, 0.75–1.32; P = 0.992) were not associated with AKI. Similarly, no significant differences were observed between mild AKI (OR, 1.00; 95% CI, 0.00–Inf; P = 1.000) and severe AKI (OR, 1.00; 95% CI, 0.00–Inf; P = 1.000). Stratification by surgery type also showed no significant associations for aortic surgery (OR, 0.89; 95% CI, 0.58–1.37; P = 0.599), coronary bypass (OR, 1.07; 95% CI, 0.78–1.47; P = 0.681), valve surgery (OR, 1.22; 95% CI, 0.81–1.85; P = 0.346), or combined surgery (OR, 0.60; 95% CI, 0.22–1.64; P = 0.320). These findings suggest that the lack of association between statin therapy and AKI is consistent across different doses, AKI severity levels, and types of cardiac surgery.

### 3.5 Kaplan-Meier analysis

**FIGURE 5:**
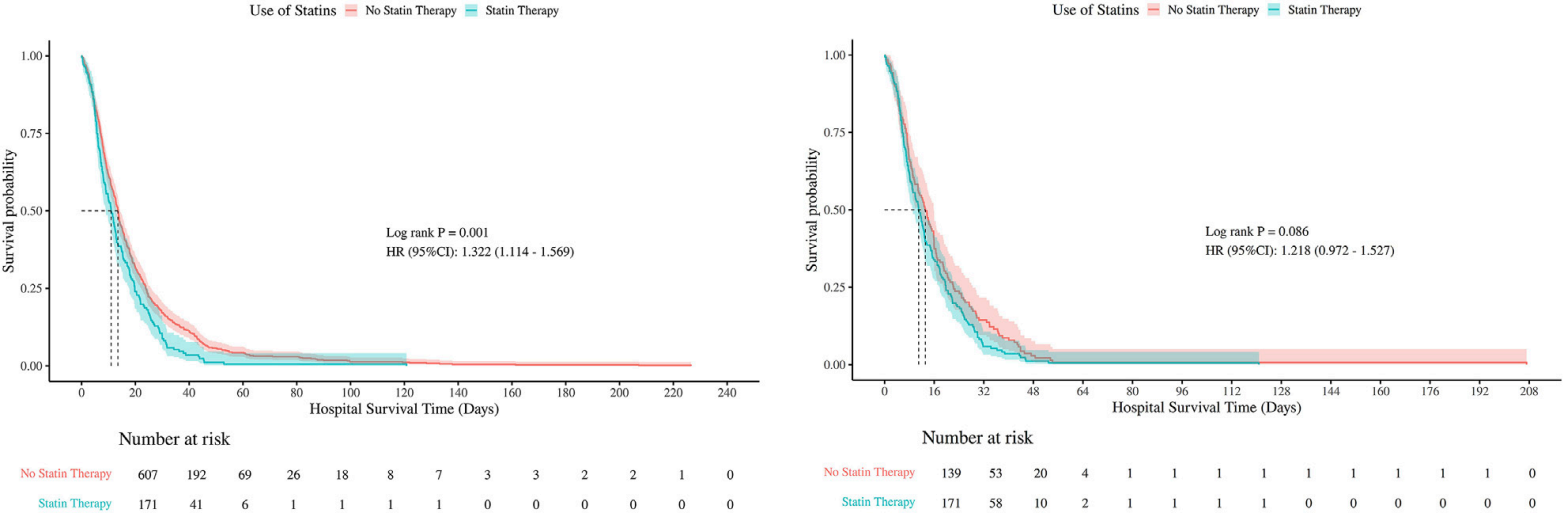
Kaplan-Meier curves for in-hospital survival (Left: Before PSM; Right: After PSM).

**FIGURE 6:**
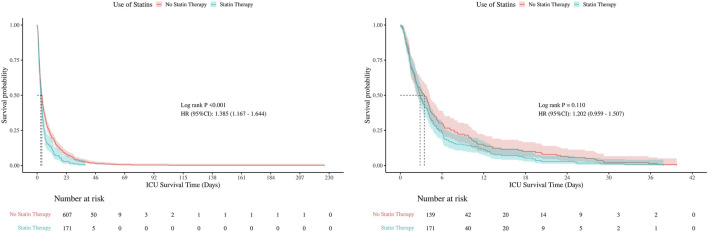
Kaplan-Meier curves for ICU survival (Left: Before PSM; Right: After PSM).

Kaplan-Meier survival curves were used to compare hospital and ICU survival between statin users and non-users, before and after PSM (Figures 5, 6). Before PSM, statin users showed slightly lower survival rates (P < 0.05), but after PSM, the curves converged, indicating no significant difference (P > 0.05). This suggests that initial survival differences were likely due to confounding factors, not statin use.

**FIGURE 7:**
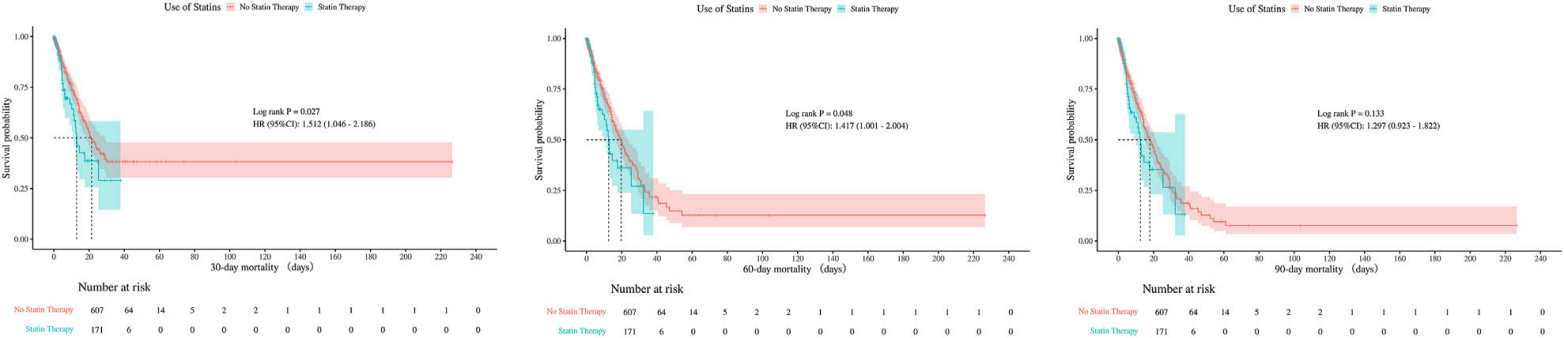
Kaplan-Meier survival curves for ICU mortality at 30, 60, and 90 Days before PSM.

**FIGURE 8:**
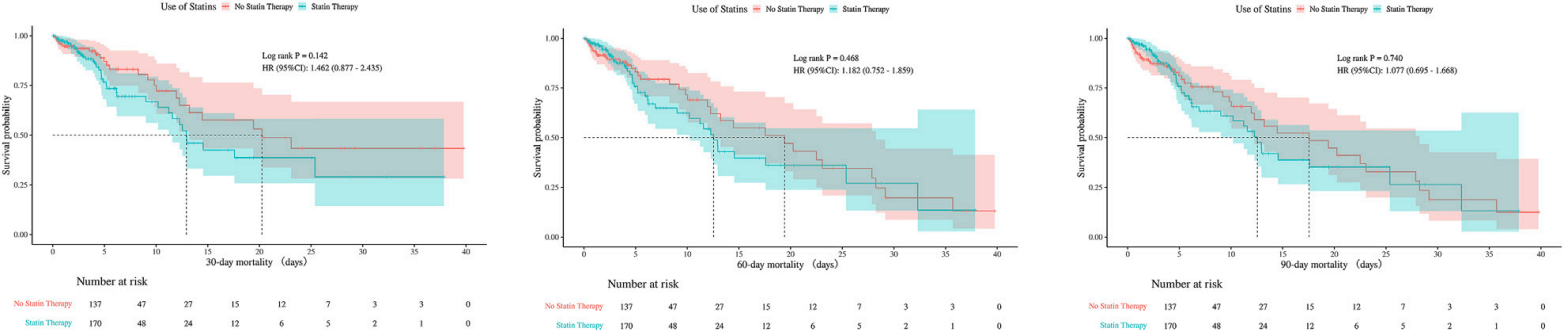
Kaplan-Meier survival curves for ICU mortality at 30, 60, and 90 Days after PSM.

There were no statistically significant differences in 30-day, 60-day, or 90-day mortality between groups, neither before nor after PSM (all P > 0.05). The consistent null findings across all time points—despite balancing baseline confounders through PSM—indicate that preexisting statin exposure was not associated with a reduction in short- or mid-term mortality within this cohort (Table 1). Although before PSM analysis indicated statistically significant differences in ICU mortality at 30- and 60-days (P < 0.05), these differences were no longer significant after PSM, with all P-values exceeding 0.05 at 30, 60, and 90 days post-matching (Figures 7, 8).

## 4 Discussion

In this retrospective cohort study, we investigated the association between preexisting statin use and the risk of AKI in patients undergoing cardiac surgery. Our findings suggest that preexisting statin use was not significantly associated with a reduced risk of AKI, RRT incidence, duration of RRT, in-hospital mortality, in-ICU mortality, 30-day mortality, 60-day mortality, or 90-day mortality after adjusting for potential confounders (all P-values were greater than 0.05). These results were consistent across multiple sensitivity analyses, including PSM and subgroup analyses, indicating the robustness of our findings.Although the MIMIC database lacks direct measures of renal recovery, shorter durations of RRT are generally associated with more rapid improvement in renal function in clinical practice. However, our study found no significant association between preoperative statin use and renal recovery in AKI patients (RRT duration P-values were above 0.05). The median 7-day preexisting statin exposure observed in our study exceeds the 3–5 days generally considered necessary for pleiotropic effects, indicating that insufficient treatment duration is unlikely to explain our negative findings. In the existing literature, treatment duration may significantly influence the incidence of AKI (Prowle et al., 2012). Additionally, the absence of renal protection against AKI despite this adequate exposure further questions the benefit of preexisting statin loading initiated 1 week prior to surgery. These results cast doubt on the clinical relevance of statins’ purported renal protective effects in the surgical setting.

There is some heterogeneity between the results of this study and the existing literature. Brunelli et al. (2012) reported that preexisting statin use reduced the risk of AKI, whereas no significant association was found in our study. This discrepancy may stem from differences in the criteria for defining AKI (RIFLE vs. KDIGO), the types of cardiac surgery studied, and the statistical methods employed. Previous meta-analyses by Wang et al. (2015) and Li et al. (2016) suggested that perioperative statin therapy (PST) could be effective in preventing cardiac CSA-AKI. However, more recent meta-analyses have failed to demonstrate a significant impact of statins on renal outcomes. For example, Wang et al. (2017) reported that perioperative statin use might even adversely affect short-term renal outcomes in cardiac surgery patients, although the incidence of severe renal complications remained unchanged. Similarly, Zhen-Han et al. (2017) concluded that statins had no effect on AKI or myocardial infarction following cardiac surgery. Notably, several high-quality studies support our findings, including a randomized controlled trial (RCT) by Park et al. (2016), which confirmed that statins do not reduce the incidence of AKI after heart valve surgery. An RCT-only meta-analysis (Lewicki et al., 2015) involving only prospective randomized controlled studies evaluating the efficacy of statins in preserving major organ function after cardiac surgery concluded that preexisting statin use did not affect the incidence of postoperative AKI. An observational study by Argalious et al. (2010), based on 11,000 patients, similarly found no significant difference in the association between preexisting statin use and AKI in patients undergoing CABG or valve surgery; this is supported by other relevant studies (Nemati and Astaneh, 2015; Bolesta et al., 2011), with a studie indicating that preexisting statin administration even be associated with an elevated AKI risk in cardiac surgical patients (Putzu et al., 2016). While the database limitations preclude assessment of long-term statin use prior to surgery, existing evidence suggests that initiating statin therapy immediately before cardiac surgery provide no clinical benefit and could potentially be harmful (Romagnoli and Ricci, 2016). Despite the differences in the types of cardiac surgery explored in previous studies and the inclusion of covariates, none have confirmed an association between statins and the development of AKI. The strengths of our study include the use of a large, diverse cohort (n = 4,783), rigorous PSM analysis with multifactorial correction, and comprehensive sensitivity analyses, which provide a reliable, evidence-based foundation for clinical decision-making.

Regarding secondary outcomes, there was significant heterogeneity in the results of available studies. Shishi et al.’s (Li et al., 2023) study based on the MIMIC-IV database reported that statins reduced in-hospital and 30-day mortality rates, contradicting our findings. This discrepancy may have arisen from differences in database versions (version 2.0) and covariate adjustment strategies, as their study did not specify the type of cardiac surgery. Notably, the negative results of our study are consistent with the findings of Ali et al., who similarly did not observe a significant association between mortality and statin use in patients undergoing CABG or valve surgery. These conflicting findings suggest that the impact of statins on the prognosis of patients undergoing cardiac surgery may be moderated by multiple factors, including study design, population characteristics, and statistical methods. Further large-sample studies are needed to elucidate the effect of statins on mortality.

In existing studies, high-dose perioperative statin therapy has not been shown to reduce the overall risk of AKI, either in patients not receiving statins or in those already on statin therapy (Billings et al., 2016), even with some evidence suggesting potential adverse effects on renal function at higher doses (Bangalore et al., 2014). While our study and the work of Mithani et al. (2011) found no association between preexisting low or high dose statin use and postoperative AKI incidence, these findings collectively highlight the need to optimize statin dosing to maximize potential benefits while minimizing adverse effects. In our subgroup analyses, we further examined the effects of statin use across different AKI severities and surgical types. In the subgroup analysis, OR (95% CI) was reported as 1.00 (0.00–Inf) across different AKI stages before and PSM. This result arises from complete data separation: in this subgroup, all patients, regardless of statin therapy, experienced the endpoint event, resulting in a 100% incidence in both groups. Such a scenario caused the logistic regression model to encounter the complete separation problem, inhibiting the calculation of valid ORs and rendering statistical comparisons impossible. Potential reasons include clinical homogeneity and limited subgroup sample sizes, which reduce statistical power to detect between-group differences. Notably, no significant interactions were also observed in the subgroups divided for type of surgery, indicating that the lack of association between statin use and AKI risk is consistent across all subgroups, suggesting that statin therapy may not provide a protective effect against AKI in cardiac surgery patients.

Despite the significant pleiotropic effects of statins, such as anti-inflammatory properties, antioxidant effects, improvement of endothelial function, and plaque stabilization (Colhoun et al., 2004), this study found that preexisting statin therapy did not significantly reduce the incidence of AKI after cardiac surgery. Mechanistically, statins exert potential renoprotective effects through multiple pathways, including reducing reactive oxygen species (ROS) generation, upregulating endothelial-type nitric oxide synthase (eNOS) activity, and inhibiting angiotensin II and endothelin expression to improve endothelial function (Verma et al., 2004; Laufs et al., 1997; Lefer et al., 2001) attenuating inflammatory responses by modulating lymphocyte activation (Kwak et al., 2000); and reducing the inflammatory response through inhibition of platelet aggregation and promotion of tissue plasminogen activator (tPA) synthesis to reduce thrombosis (Dávila-Román et al., 1999). These mechanisms should theoretically attenuate AKI risk factors such as ischemia-reperfusion injury, systemic inflammatory response, and renal embolism. However, cardiac surgery-induced AKI involves more complex pathophysiologic processes (Kamla et al., 2025), especially the damaging effects of the intense systemic inflammatory response triggered by extracorporeal circulation (CPB), unavoidable ischemia-reperfusion injury, prolonged surgical time, and intraoperative hypotension, which may far outweigh the protective effects that statins can provide. Additionally, differences in statin use regimens (including dose, duration, and timing of administration) in clinical practice may also contribute to the inconsistent results across studies. These findings suggest that although basic studies have confirmed the renoprotective mechanisms of statins, the strong pathophysiologic changes caused by cardiac surgery may completely mask their theoretical protective effects, resulting in no significant effect on the incidence of postoperative AKI.

Despite the comprehensive analysis presented in our study, several limitations persist. First, as a retrospective cohort study, our findings may be subject to inherent biases, including residual confounding and unmeasured variables (e.g., socioeconomic status, baseline inflammatory markers, medication adherence), particularly given the significant impact of statins on patients at high cardiovascular risk (Allou et al., 2010) and those with advanced kidney disease (Rysz et al., 2009). Additionally, although this study included patients undergoing various types of cardiac surgeries (including CABG, valvular, and aortic procedures), several key clinical parameters could not be obtained due to systematic limitations of the MIMIC database. Specifically, intraoperative variables such as extracorporeal circulation time, aortic cross-clamp duration, surgical technique, intraoperative hypotensive events, blood transfusion volume, and operative duration were unavailable. Furthermore, important factors influencing AKI risk—such as iodinated contrast use, inflammatory markers (e.g., C-reactive protein and calcitoninogen), and the urgency of the procedure (elective vs. emergency)—were not captured. These indicators are critical in the pathophysiology of cardiac surgery-CSA-AKI. The absence of such data may result in residual confounding, which is a common challenge in ICU database studies. Although we employed PSM to mitigate confounding, uncontrolled confounders, such as the use of other medications (e.g., beta-blockers, calcium channel blockers) and changes in surgical and anesthetic practices over time, may still influence our results. Second, the findings derived from the U.S. single-center database (MIMIC-IV) may be affected by the specificity of the healthcare system, necessitating caution when extrapolating to other regional populations. Additionally, we were unable to ascertain whether patients received statins preoperatively, which could impact the interpretation of our results, particularly in those not treated with statins, as short-term perioperative use may yield different outcomes (Billings et al., 2016; Patti et al., 2011). Treatment should not be initiated solely to protect renal function. Notably, variations in baseline risk profiles, pharmacokinetics, and pharmacodynamics of statins across populations warrant attention (Yang et al., 2024). Future prospective studies, particularly multicenter randomized controlled trials, are essential to validate our findings in a broader and more diverse population and to further elucidate the potential mechanisms and clinical applicability of statins in preventing AKI following cardiac surgery.

Despite these limitations, our findings carry important clinical implications. They suggest that routine preexisting use of statins may not effectively prevent cardiac surgery-related AKI, a conclusion with significant ramifications for clinical practice. Based on the available evidence, we recommend that clinicians prioritize AKI prevention and treatment strategies supported by evidence-based medicine, including optimization of hemodynamic management, judicious avoidance of nephrotoxic medications, and individualized volumetric therapy regimens. Future studies should further investigate the potential benefits of statins in specific patient subgroups (e.g., those with chronic kidney disease or high-risk surgical patients) and assess the effects of varying dosing regimens (e.g., long-term therapy or intensive perioperative therapy). Existing studies utilizing animal models have demonstrated reduced tissue damage when statins are administered 24 h prior to planned experimental injury (Yao et al., 2006). RRT is a vital intervention for severe AKI. Available studies suggest that preexisting statin use may be associated with a decreased need for RRT, a potential relationship that warrants further investigation in future research (Huffmyer et al., 2009). Investigating the early postoperative use of statins on AKI incidence may also provide insights for clinical dosing (Billings et al., 2016; Billings et al., 2010). Additionally, research should focus on other pharmacologic interventions that may mitigate the risk of AKI in patients undergoing cardiac surgery to furnish more evidence-based guidance for clinical practice.

## 5 Conclusion

Our study found no significant association between preexisting statin use and a reduced risk of AKI, in-hospital mortality, or in-ICU mortality among patients undergoing cardiac surgery. Similarly, no significant associations were observed for 30-day, 60-day, or 90-day mortality outcomes. Given the pleiotropic effects of statins and their established safety profile, future research should aim to confirm these findings through randomized controlled trials and explore the broader effects of statin therapy on various subgroups. This approach could provide a more robust evidence-based rationale for renal protection strategies in patients undergoing cardiac surgery.

## References

[B1] Allou N. Augustin P. Dufour G. Tini L. Ibrahim H. Dilly M. P. (2010). Preoperative statin treatment is associated with reduced postoperative mortality after isolated cardiac valve surgery in high-risk patients. J. Cardiothorac. Vasc. Anesth. 24 (6), 921–926. 10.1053/j.jvca.2010.03.017 20638866

[B2] Argalious M. Xu M. Sun Z. Smedira N. Koch C. G. (2010). Preoperative statin therapy is not associated with a reduced incidence of postoperative acute kidney injury after cardiac surgery. Anesth. Analg. 111 (2), 324–330. 10.1213/ANE.0b013e3181d8a078 20375302

[B3] Bangalore S. Fayyad R. Hovingh G. K. Laskey R. Vogt L. DeMicco D. A. (2014). Statin and the risk of renal-related serious adverse events: analysis from the IDEAL, TNT, CARDS, ASPEN, SPARCL, and other placebo-controlled trials. Am. J. Cardiol. 113 (12), 2018–2020. 10.1016/j.amjcard.2014.03.046 24793673

[B4] Bao Y. W. Yuan Y. Chen J. H. Lin W. Q. (2018). Kidney disease models: tools to identify mechanisms and potential therapeutic targets. Zool. Res. 39 (2), 72–86. 10.24272/j.issn.2095-8137.2017.055 29515089 PMC5885387

[B5] Bihorac A. Yavas S. Subbiah S. Hobson C. E. Schold J. D. Gabrielli A. (2009). Long-term risk of mortality and acute kidney injury during hospitalization after major surgery. Ann. Surg. 249 (5), 851–858. 10.1097/SLA.0b013e3181a40a0b 19387314

[B6] Billings F. T. Hendricks P. A. Schildcrout J. S. Shi Y. Petracek M. R. Byrne J. G. (2016). High-dose perioperative atorvastatin and acute kidney injury following cardiac surgery: a randomized clinical trial. JAMA 315 (9), 877–888. 10.1001/jama.2016.0548 26906014 PMC4843765

[B7] Billings F. T. Pretorius M. Siew E. D. Yu C. Brown N. J. (2010). Early postoperative statin therapy is associated with a lower incidence of acute kidney injury after cardiac surgery. J. Cardiothorac. Vasc. Anesth. 24 (6), 913–920. 10.1053/j.jvca.2010.03.024 20599398 PMC2992577

[B8] Bolesta S. Uhrin L. M. Guzek J. R. (2011). Preoperative statins and acute kidney injury after cardiac surgery: utilization of a consensus definition of acute kidney injury. Ann. Pharmacother. 45 (1), 23–30. 10.1345/aph.1P384 21205946

[B9] Brouilette S. W. Moore J. S. McMahon A. D. Thompson J. R. Ford I. Shepherd J. (2007). Telomere length, risk of coronary heart disease, and statin treatment in the West of Scotland Primary Prevention Study: a nested case-control study. Lancet 369 (9556), 107–114. 10.1016/S0140-6736(07)60071-3 17223473

[B10] Brunelli S. M. Waikar S. S. Bateman B. T. Chang T. I. Lii J. Garg A. X. (2012). Preoperative statin use and postoperative acute kidney injury. Am. J. Med. 125 (12), 1195–1204. 10.1016/j.amjmed.2012.06.021 23062398 PMC3597342

[B11] Chertow G. M. Burdick E. Honour M. Bonventre J. V. Bates D. W. (2005). Acute kidney injury, mortality, length of stay, and costs in hospitalized patients. J. Am. Soc. Nephrol. 16 (11), 3365–3370. 10.1681/ASN.2004090740 16177006

[B12] Chertow G. M. Levy E. M. Hammermeister K. E. Grover F. Daley J. (1998). Independent association between acute renal failure and mortality following cardiac surgery. Am. J. Med. 104 (4), 343–348. 10.1016/s0002-9343(98)00058-8 9576407

[B13] Chou R. Cantor A. Dana T. Wagner J. Ahmed A. Fu R. (2022). Statin use for the primary prevention of cardiovascular disease in adults: a systematic review for the U.S. Preventive services task force. Rockville, MD: Agency for Healthcare Research and Quality US. 36067345

[B14] Colhoun H. M. Betteridge D. J. Durrington P. N. Hitman G. A. Neil H. A. Livingstone S. J. (2004). Primary prevention of cardiovascular disease with atorvastatin in type 2 diabetes in the Collaborative Atorvastatin Diabetes Study (CARDS): multicentre randomised placebo-controlled trial. Lancet 364 (9435), 685–696. 10.1016/S0140-6736(04)16895-5 15325833

[B15] Dávila-Román V. G. Kouchoukos N. T. Schechtman K. B. Barzilai B. (1999). Atherosclerosis of the ascending aorta is a predictor of renal dysfunction after cardiac operations. J. Thorac. Cardiovasc Surg. 117 (1), 111–116. 10.1016/s0022-5223(99)70475-7 9869764

[B16] Greenwood J. Steinman L. Zamvil S. S. (2006). Statin therapy and autoimmune disease: from protein prenylation to immunomodulation. Nat. Rev. Immunol. 6 (5), 358–370. 10.1038/nri1839 16639429 PMC3842637

[B17] Gueler F. Rong S. Park J. K. Fiebeler A. Menne J. Elger M. (2002). Postischemic acute renal failure is reduced by short-term statin treatment in a rat model. J. Am. Soc. Nephrol. 13 (9), 2288–2298. 10.1097/01.asn.0000026609.45827.3d 12191973

[B18] Huffmyer J. L. Mauermann W. J. Thiele R. H. Ma J. Z. Nemergut E. C. (2009). Preoperative statin administration is associated with lower mortality and decreased need for postoperative hemodialysis in patients undergoing coronary artery bypass graft surgery. J. Cardiothorac. Vasc. Anesth. 23 (4), 468–473. 10.1053/j.jvca.2008.11.005 19157909

[B19] Johnson A. E. W. Bulgarelli L. Shen L. Gayles A. Shammout A. Horng S. (2023). MIMIC-IV, a freely accessible electronic health record dataset. Sci. Data 10, 1. 10.1038/s41597-022-01899-x 36596836 PMC9810617

[B20] Kamla C. E. Meersch-Dini M. Palma L. M. P. (2025). Kidney injury following cardiac surgery: a review of our current understanding. Am. J. Cardiovasc Drugs 25, 337–348. 10.1007/s40256-024-00715-8 39799538 PMC12014718

[B21] Kivipelto M. Solomon A. Winblad B. (2005). Statin therapy in Alzheimer's disease. Lancet Neurol. 4 (9), 521–522. 10.1016/S1474-4422(05)70150-2 16109358

[B22] Kwak B. Mulhaupt F. Myit S. Mach F. (2000). Statins as a newly recognized type of immunomodulator. Nat. Med. 6 (12), 1399–1402. 10.1038/82219 11100127

[B23] Laufs U. Fata V. L. Liao J. K. (1997). Inhibition of 3-hydroxy-3-methylglutaryl (HMG)-CoA reductase blocks hypoxia-mediated down-regulation of endothelial nitric oxide synthase. J. Biol. Chem. 272 (50), 31725–31729. 10.1074/jbc.272.50.31725 9395516

[B24] Lefer A. M. Scalia R. Lefer D. J. (2001). Vascular effects of HMG CoA-reductase inhibitors (statins) unrelated to cholesterol lowering: new concepts for cardiovascular disease. Cardiovasc Res. 49 (2), 281–287. 10.1016/s0008-6363(00)00247-9 11164838

[B25] Lewicki M. Ng I. Schneider A. G. (2015). HMG CoA reductase inhibitors (statins) for preventing acute kidney injury after surgical procedures requiring cardiac bypass. Cochrane Database Syst. Rev. 2015 (3), CD010480. 10.1002/14651858.CD010480.pub2 25758322 PMC10788137

[B26] Li M. Zou H. Xu G. (2016). The prevention of statins against AKI and mortality following cardiac surgery: a meta-analysis. Int. J. Cardiol. 222, 260–266. 10.1016/j.ijcard.2016.07.173 27497107

[B27] Li S. Zhang Y. Yang Y. Chen S. Yang Z. Kuang C. (2023). The impact of statin use before intensive care unit admission on patients with acute kidney injury after cardiac surgery. Front. Pharmacol. 14, 1259828. 10.3389/fphar.2023.1259828 37781714 PMC10537929

[B28] Li S. Y. Chen J. Y. Yang W. C. Chuang C. L. (2011). Acute kidney injury network classification predicts in-hospital and long-term mortality in patients undergoing elective coronary artery bypass grafting surgery. Eur. J. Cardiothorac. Surg. 39 (3), 323–328. 10.1016/j.ejcts.2010.07.010 20739188

[B29] López-Miranda J. Pedro-Botet J. (2021). Therapeutic targets in the treatment of dyslipidaemias: from statins to PCSK9 inhibitors. Unmet needs. Clin. Investig. Arterioscler. 33 (Suppl. 1), 46–52. 10.1016/j.arteri.2020.12.005 33966813

[B30] Macedo E. Mehta R. L. (2015). Preventing acute kidney injury. Crit. Care Clin. 31 (4), 773–784. 10.1016/j.ccc.2015.06.011 26410144

[B31] Mithani S. Kuskowski M. Slinin Y. Ishani A. McFalls E. Adabag S. (2011). Dose-dependent effect of statins on the incidence of acute kidney injury after cardiac surgery. Ann. Thorac. Surg. 91 (2), 520–525. 10.1016/j.athoracsur.2010.10.061 21256305

[B32] Molnar A. O. Parikh C. R. Coca S. G. Thiessen-Philbrook H. Koyner J. L. Shlipak M. G. (2014). Association between preoperative statin use and acute kidney injury biomarkers in cardiac surgical procedures. Ann. Thorac. Surg. 97 (6), 2081–2087. 10.1016/j.athoracsur.2014.02.033 24725831 PMC4068122

[B33] Nemati M. H. Astaneh B. (2015). The effects of preoperative statins on the incidence of postoperative acute kidney injury in patients undergoing cardiac surgeries. Interact. Cardiovasc Thorac. Surg. 21 (4), 493–498. 10.1093/icvts/ivv194 26180093

[B34] Novack V. Terblanche M. Almog Y. (2006). Do statins have a role in preventing or treating sepsis? Crit. Care 10 (1), 113. 10.1186/cc3972 16469122 PMC1550787

[B35] Park J. H. Shim J. K. Song J. W. Soh S. Kwak Y. L. (2016). Effect of atorvastatin on the incidence of acute kidney injury following valvular heart surgery: a randomized, placebo-controlled trial. Intensive Care Med. 42 (9), 1398–1407. 10.1007/s00134-016-4358-8 27120082

[B36] Patti G. Ricottini E. Nusca A. Colonna G. Pasceri V. D'Ambrosio A. (2011). Short-term, high-dose Atorvastatin pretreatment to prevent contrast-induced nephropathy in patients with acute coronary syndromes undergoing percutaneous coronary intervention from the ARMYDA-CIN [atorvastatin for reduction of myocardial damage during angioplasty--contrast-induced nephropathy] trial. Am. J. Cardiol. 108 (1), 1–7. 10.1016/j.amjcard.2011.03.001 21529740

[B37] Priyanka P. Zarbock A. Izawa J. Gleason T. G. Renfurm R. W. Kellum J. A. (2021). The impact of acute kidney injury by serum creatinine or urine output criteria on major adverse kidney events in cardiac surgery patients. J. Thorac. Cardiovasc Surg. 162 (1), 143–151.e7. 10.1016/j.jtcvs.2019.11.137 32033818

[B38] Prowle J. R. Calzavacca P. Licari E. Ligabo E. V. Echeverri J. E. Haase M. (2012). Pilot double-blind, randomized controlled trial of short-term atorvastatin for prevention of acute kidney injury after cardiac surgery. Nephrol. Carlt. 17 (3), 215–224. 10.1111/j.1440-1797.2011.01546.x 22117606

[B39] Putzu A. Capelli B. Belletti A. Cassina T. Ferrari E. Gallo M. (2016). Perioperative statin therapy in cardiac surgery: a meta-analysis of randomized controlled trials. Crit. Care 20 (1), 395. 10.1186/s13054-016-1560-6 27919293 PMC5139027

[B40] Ray S. (2024). Role of statins in the management of dyslipidaemia. Indian Heart J. 76 (Suppl. 1), S33–S37. 10.1016/j.ihj.2023.11.267 38599727 PMC11019333

[B41] Romagnoli S. Ricci Z. (2016). Statins and acute kidney injury following cardiac surgery: has the last word been told? J. Thorac. Dis. 8 (6), E451–E454. 10.21037/jtd.2016.04.34 27294251 PMC4885980

[B42] Rosenson R. S. (2006). Low high-density lipoprotein cholesterol and cardiovascular disease: risk reduction with statin therapy. Am. Heart J. 151 (3), 556–563. 10.1016/j.ahj.2005.03.049 16504615

[B43] Rysz J. Aronow W. S. Stolarek R. S. Hannam S. Mikhailidis D. P. Banach M. (2009). Nephroprotective and clinical potential of statins in dialyzed patients. Expert Opin. Ther. Targets 13 (5), 541–550. 10.1517/14728220902882130 19368496

[B44] Sabbatini M. Pisani A. Uccello F. Serio V. Serù R. Paternò R. (2004). Atorvastatin improves the course of ischemic acute renal failure in aging rats. J. Am. Soc. Nephrol. 15 (4), 901–909. 10.1097/01.asn.0000119573.01290.ae 15034092

[B45] Safarova M. S. Weintraub S. Sadaniantz K. Kovell L. Warden B. A. Garshick M. S. (2025). Statin use in special populations for the prevention of cardiovascular disease in adults. Curr. Atheroscler. Rep. 27 (1), 54. 10.1007/s11883-025-01298-8 40310600

[B46] Santodomingo-Garzón T. Cunha T. M. Verri W. A. Jr Valério D. A. Parada C. A. Poole S. (2006). Atorvastatin inhibits inflammatory hypernociception. Br. J. Pharmacol. 149 (1), 14–22. 10.1038/sj.bjp.0706836 16865092 PMC1629407

[B47] Teo S. H. Endre Z. H. (2017). Biomarkers in acute kidney injury (AKI). Best. Pract. Res. Clin. Anaesthesiol. 31 (3), 331–344. 10.1016/j.bpa.2017.10.003 29248140

[B48] Terblanche M. Almog Y. Rosenson R. S. Smith T. S. Hackam D. G. (2007). Statins and sepsis: multiple modifications at multiple levels. Lancet Infect. Dis. 7 (5), 358–368. 10.1016/S1473-3099(07)70111-1 17448939

[B49] Tu B. Tang Y. Cheng Y. Yang Y. Wu C. Liu X. (2021). Association of prior to intensive care unit statin use with outcomes on patients with acute kidney injury. Front. Med. (Lausanne) 8, 810651. 10.3389/fmed.2021.810651 35004788 PMC8739269

[B50] Verma S. Rao V. Weisel R. D. Li S. H. Fedak P. W. Miriuka S. (2004). Novel cardioprotective effects of pravastatin in human ventricular cardiomyocytes subjected to hypoxia and reoxygenation: beneficial effects of statins independent of endothelial cells. J. Surg. Res. 119 (1), 66–71. 10.1016/j.jss.2003.10.011 15126084

[B51] Wang C. Gao Y. Tian Y. Wang Y. Zhao W. Sessler D. I. (2021). Prediction of acute kidney injury after cardiac surgery from preoperative N-terminal pro-B-type natriuretic peptide. Br. J. Anaesth. 127 (6), 862–870. 10.1016/j.bja.2021.08.015 34561052

[B52] Wang C. Y. Liu P. Y. Liao J. K. (2008). Pleiotropic effects of statin therapy: molecular mechanisms and clinical results. Trends Mol. Med. 14 (1), 37–44. 10.1016/j.molmed.2007.11.004 18068482 PMC2621332

[B53] Wang J. Gu C. Gao M. Yu W. Yu Y. (2015). Preoperative statin therapy and renal outcomes after cardiac surgery: a meta-analysis and meta-regression of 59,771 patients. Can. J. Cardiol. 31 (8), 1051–1060. 10.1016/j.cjca.2015.02.034 26081692

[B54] Wang S. Yao H. Yu H. Chen C. Zhou R. Wang R. (2017). Effect of perioperative statin therapy on renal outcome in patients undergoing cardiac surgery: a meta-analysis of randomized controlled trials. Med. Baltim. 96 (19), e6883. 10.1097/MD.0000000000006883 PMC5428625 28489791

[B55] Wang Y. Bellomo R. (2017). Cardiac surgery-associated acute kidney injury: risk factors, pathophysiology and treatment. Nat. Rev. Nephrol. 13 (11), 697–711. 10.1038/nrneph.2017.119 28869251

[B56] Yang A. B. Mhango G. Kong C. Y. Lin J. J. Wisnivesky J. P. Leiter A. (2024). Statin prescription disparities in patients with breast cancer and diabetes for primary cardiovascular disease prevention. Front. Oncol. 14, 1483918. 10.3389/fonc.2024.1483918 39568566 PMC11576278

[B57] Yao H. W. Mao L. G. Zhu J. P. (2006). Protective effects of pravastatin in murine lipopolysaccharide-induced acute lung injury. Clin. Exp. Pharmacol. Physiol. 33 (9), 793–797. 10.1111/j.1440-1681.2006.04440.x 16922808

[B58] Zheng Z. Jayaram R. Jiang L. Emberson J. Zhao Y. Li Q. (2016). Perioperative rosuvastatin in cardiac surgery. N. Engl. J. Med. 374 (18), 1744–1753. 10.1056/NEJMoa1507750 27144849

[B59] Zhen-Han L. Rui S. Dan C. Xiao-Li Z. Qing-Chen W. Bo F. (2017). Perioperative statin administration with decreased risk of postoperative atrial fibrillation, but not acute kidney injury or myocardial infarction: a meta-analysis. Sci. Rep. 7 (1), 10091. 10.1038/s41598-017-10600-x 28855628 PMC5577099

[B60] Zou C. Wang C. Lu L. (2022). Advances in the study of subclinical AKI biomarkers. Front. Physiol. 13, 960059. 10.3389/fphys.2022.960059 36091391 PMC9449362

