# Correction: Preexisting statin therapy is not associated with reduced acute kidney injury following cardiac surgery: a retrospective analysis

**DOI:** 10.3389/fphar.2025.1657887

**Published:** 2025-08-19

**Authors:** Jia Wang, Chuzhu Huang, Yan Chen, Yilin Huang, Zhuomin Wu

**Affiliations:** The First Affiliated Hospital of Shantou University School of Medicine, Shantou, China

**Keywords:** CSA-AKI, statins, MIMIC-IV, cardiac surgery, AKI

The published article was incorrectly categorized as a Review article. The correct article type is “Original Research”.

The original version of this article has been updated.

