# Supplementary material for: Preexisting statin therapy is not associated with reduced acute kidney injury following cardiac surgery: a retrospective analysis
Source: Front Pharmacol. 2025 May 30;16:1613681. doi: 10.3389/fphar.2025.1613681 (PMC12162650; doi:10.3389/fphar.2025.1613681)
Supplement: Supplementary file 1 [file Table1.docx]

Supplementary Table： Statin Equivalent Dose

| Statins | Common Dose (mg) | Equivalent Simvastatin Dose (mg) |
| --- | --- | --- |
| Simvastatin | 10 | 10 |
|  | 20 | 20 |
|  | 40 | 40 |
|  | 80 | 80 |
| Pravastatin | 10 | 5 |
|  | 20 | 10 |
|  | 40 | 20 |
|  | 80 | 40 |
| Atorvastatin | 10 | 20 |
|  | 20 | 40 |
|  | 40 | 80 |
|  | 80 | - (no equivalent) |
| Rosuvastatin | 5 | 20 |
|  | 10 | 40 |
|  | 20 | 80 |
